# Comprehensive analysis of macrophage-related genes in prostate cancer by integrated analysis of single-cell and bulk RNA sequencing

**DOI:** 10.18632/aging.205727

**Published:** 2024-04-24

**Authors:** Jili Zhang, Zhihao Li, Zhenlin Chen, Wenzhen Shi, Yue Xu, Zhangcheng Huang, Zequn Lin, Ruiling Dou, Shaoshan Lin, Xin Jiang, Mengqiang Li, Shaoqin Jiang

**Affiliations:** 1 Department of Urology, Fujian Union Hospital, Fujian Medical University, Fuzhou, Fujian, China; 2 Department of Urology, The First Navy Hospital of Southern Theater Command, Zhanjiang, Guangdong, China; 3 Center of Reproductive Medicine, Fujian Maternity and Child Health Hospital, Fujian Medical University, Fuzhou, Fujian, China

**Keywords:** prostate cancer, macrophage, single-cell RNA-sequencing, unsupervised clustering, tumor microenvironment

## Abstract

Macrophages, as essential components of the tumor immune microenvironment (TIME), could promote growth and invasion in many cancers. However, the role of macrophages in tumor microenvironment (TME) and immunotherapy in PCa is largely unexplored at present. Here, we investigated the roles of macrophage-related genes in molecular stratification, prognosis, TME, and immunotherapeutic response in PCa. Public databases provided single-cell RNA sequencing (scRNA-seq) and bulk RNAseq data. Using the Seurat R package, scRNA-seq data was processed and macrophage clusters were identified automatically and manually. Using the CellChat R package, intercellular communication analysis revealed that tumor-associated macrophages (TAMs) interact with other cells in the PCa TME primarily through MIF - (CD74+CXCR4) and MIF - (CD74+CD44) ligand-receptor pairs. We constructed coexpression networks of macrophages using the WGCNA to identify macrophage-related genes. Using the R package ConsensusClusterPlus, unsupervised hierarchical clustering analysis identified two distinct macrophage-associated subtypes, which have significantly different pathway activation status, TIME, and immunotherapeutic efficacy. Next, an 8-gene macrophage-related risk signature (MRS) was established through the LASSO Cox regression analysis with 10-fold cross-validation, and the performance of the MRS was validated in eight external PCa cohorts. The high-risk group had more active immune-related functions, more infiltrating immune cells, higher HLA and immune checkpoint gene expression, higher immune scores, and lower TIDE scores. Finally, the NCF4 gene has been identified as the hub gene in MRS using the “mgeneSim” function.

## INTRODUCTION

Prostate cancer (PCa) is the most prevalent tumor among men in Western nations, whose morbidity has been on the rise in recent years, accounting for 27% of newly detected tumor cases in men, and is also the second leading killer of cancer death [1]. For clinically localized prostate cancer, radical prostatectomy (RP) is still the most commonly used standard treatment option [2–4]. Unfortunately, many patients continue to progress to metastatic prostate cancer even after successful RP therapy [5, 6]. The 5-year survival rate for PCa patients with distant metastases is approximately 30% [7]. Therefore, early detection of disease progression in patients is key to reducing PCa mortality. However, PCa is a heterogeneous disease with a diverse prognosis, and the combination of clinical and pathological information does not fully elucidate tumor behavior [8]. In addition, it is not reliable to predict disease progression using frequently used clinical factors, including prostate-specific antigen (PSA), Gleason score, and TNM staging [9]. Therefore, exploring valid and reliable biomarkers for predicting disease progression in PCa, thereby stratifying PCa patients for risk of disease progression, and then exploring sensitive treatment options for high-risk patients, will help urologists to make rational clinical decisions and aggressively individualize treatments for patients in different risk strata, thereby improving prognosis. Additionally, the molecular mechanisms responsible for tumor progression could be clarified and novel therapeutic targets could be developed based on these biomarkers.

Tumor progression and invasion are influenced by the TME, which contains a variety of cell types, including immune cells, stromal cells, endothelial cells, and fibroblasts [10, 11]. TME has proven to be a highly promising therapeutic target by numerous studies demonstrating how immune compositions regulate cancer progression [12–14]. A major focus of anti-tumor immunity is the adaptive T-cell response, whereas innate immune cells still receive insufficient attention [15]. There is evidence that the TME interacts with innate immune cells to promote tumor growth [16]. Macrophages, as classical innate immune cells, have an integral role in homeostasis and immunity for healthy people but lose their protective function in the context of cancer and become Tumor-associated macrophages (TAMs), providing a favorable microenvironment for cancer growth and invasion at primary and metastatic sites [17]. In non-small cell lung cancer, the majority of immune cells in TME are TAMs [18, 19]. There are two main categories of TAMs: M1 and M2 [20, 21]. The M1 pro-inflammatory phenotype of TAMs is mainly observed in the early stage of tumor development and plays a crucial role in the inhibition of tumor growth. During the tumor development process, TAMs progressively convert to the M2 phenotype, thereby contributing to tumor angiogenesis and immunosuppression [22, 23]. There is growing evidence that dynamic changes in macrophage phenotypes play a significant role in the tumorigenesis, progression, and metastasis of tumors [24]. Additionally, TAMs can facilitate tumor metastasis and promote cancer angiogenesis by promoting epithelial-mesenchymal transition and secreting angiogenic growth factors, respectively [25–27]. It is also important to note that TAMs can inhibit anti-tumor immunity by interacting with different types of immune cells and immunosuppressive cells within the TME, thus promoting tumorigenesis [28, 29]. Therefore, it is essential to elucidate the properties of TAMs and identify biomarkers that are associated with macrophage infiltration in the treatment, prognosis, and immune infiltration mechanism of PCa. Nevertheless, the characterization of the immune microenvironment and immune cells in PCa, especially macrophages, has rarely been comprehensively and systematically studied so far.

Unlike bulk RNA sequencing (bulk RNA-seq), which focuses primarily on the average expression level of all cells in a sample and is unable to uncover the heterogeneity among tumor cells in a sample, single-cell RNA-sequencing (scRNA-seq) can identify intra-tumor heterogeneity at the cellular level [30]. With the development of scRNA-seq technology and corresponding data analysis methods, it is now possible to determine the molecular characteristics of various immune cell populations within the TME, offering a new approach to identifying functional biomarkers [31, 32]. Due to the advantage of scRNA-seq, a number of novel biomarkers for cancer have been identified using the organic combination of scRNA-seq with bulk RNA-seq [15, 33]. In this study, we identified macrophage marker genes and depicted the cell-cell communication between TAMs and other cell types within the TME on the basis of scRNA-seq analysis. Through bulk RNA-seq analysis, we constructed coexpression networks of macrophages using the weighted gene coexpression network analysis (WGCNA) to identify macrophage-related genes. Then, macrophage-related molecular subtypes with entirely different prognoses, clinicopathologic features, immune microenvironment, and immunotherapy response were identified. Next, a macrophage-related risk signature (MRS) was established for the prognosis prediction of PCa, and the performance of the MRS was validated in eight independent PCa cohorts. Meanwhile, we also revealed the dissimilarities in the immune infiltration landscape and immunotherapy between different risk groups. Moreover, we identified NCF4 as the hub gene in MRS, which may be a potential research target for PCa. Overall, these findings will serve to provide insights into how macrophages affect PCa and assist PCa patients in receiving more effective individualized treatment.

## MATERIALS AND METHODS

### Data collection

In this study, we included a total of 10 prostate cancer (PCa) cohorts, including a scRNA-seq cohort and 9 bulk RNA-seq cohorts (Supplementary Table 1). The scRNA-seq data with twelve primary prostate cancer (PCa) samples were obtained from the Gene Expression Omnibus (GEO) database (accession number GSE141445). For bulk RNA-seq data used in this study, there were nine independent PCa cohorts. First, we obtained transcriptomic profiles (Transcripts Per Kilobase Million or TPM) for 501 PCa cases and 52 normal cases from The Cancer Genome Atlas (TCGA) database (https://portal.gdc.cancer.gov/). The UCSC (University of California, Santa Cruz) Xena public data hub (https://xenabrowser.net/) provided the corresponding clinical and progression-free survival information of the TCGA cohort. The Chinese Prostate Cancer Genome and Epigenome Atlas (CPGEA, n=136) bulk RNA-seq data were downloaded from (http://www.cpgea.com/download.php), and the latest survival data were used (n=125). From the cBioPortal for Cancer Genomics (https://www.cbioportal.org/), we downloaded the bulk RNA-seq profiles and the corresponding survival data of DFKZ (The German Cancer Research Center, Deutsches Krebsforschungszentrum, n=81) and MSKCC (The Memorial Sloan Kettering Cancer Center, n=140). The GEO database (https://www.ncbi.nlm.nih.gov/geo/) was utilized to obtain the transcriptome profiles and corresponding survival data of GSE116918 (n=248), GSE70768 (n=111), GSE70769 (n=92), GSE46602 (n=36), and GSE70770 (n=203).

### scRNA-seq analysis

The R package “Seurat” was used for converting scRNA-seq data into a Seurat object [34]. The scRNA-seq data was first quality controlled by removing genes expressed in less than 3 cells, cells expressing fewer than 50 genes, and cells expressing more than 5% of mitochondrial genes. A total of 25,999 eligible cells were selected for further analysis. We then normalized the scRNA-seq data using Seurat’s “NormalizeData” function with the normalization method set to “LogNormalize”. After that, the “FindVariableFeatures” function was utilized to identify the top 1,500 highly variable genes. Following this, based on the top 1,500 genes, scRNA-seq data were reduced in dimension by performing principal component analysis (PCA) with the “RunPCA” function. The top 20 significant PCs were selected for cell clustering analysis based on the result of the JackStraw analysis. With the “Seurat” package, cell clustering analysis was conducted using the “FindNeighbors” and “FindClusters” functions. According to the PCA Euclidean distance, the “FindNeighbors” function was conducted to find the nearest neighbors of each cell and generate a k-nearest neighbor graph. The “FindClusters” function was implemented to identify clusters with a resolution of 0.5. The “RunTSNE” function was used for dimensionality reduction and visualization via t-distributed stochastic neighbor embeddings (t-SNEs). The marker genes for each cluster were identified using the “FindAllMarkers” function with the threshold set to |log2 (fold change) | > 1 and adjusted P-value < 0.05. For cluster annotation, we first used “HumanPrimaryCellAtlasData” from the R package “singleR” [35] as the reference data to assist in the annotation, and then manually annotated the different clusters using the CellMarker database [36] and the marker genes found in previous studies. For cell communication analysis, the “CellChat” R package was implemented to infer cellular communication in PCa tumor microenvironment (TME) based on receptor-ligand interactions. For the computation of communication networks, linking numbers and communication probability were calculated. A visualization was built to show how many interactions there are between any two groups of cells, as well as their strength. Visualization of the major signal sender and receiver cells through scatter plots helped to determine the largest contributors of efferent and afferent signals in the cell population.

### Macrophage co-expression network construction based on WGCNA

We estimated the relative proportion of infiltrating immune cells in each cohort using the ssGSEA algorithm. Using the R package “WGCNA”, we construct the gene co-expression networks of the TCGA-PRAD cohort (501 tumor samples), CPGEA cohort (136 tumor samples), and GSE70768 cohort (126 tumor samples) to identify co-expressed genes in macrophages. There are six main steps involved in network construction: 1. Establishment of a similarity matrix. 2. Creating an adjacency matrix from the similarity matrix using the suitable soft thresholds (TCGA=10, CPGEA=8, GSE70768=7). 3. A topological overlap matrix (TOM) was generated by transforming the adjacency matrix. 4. A hierarchical clustering tree was obtained by layering the dissTOM with Tom Cluster. 5. Modules were identified from the hierarchical clustering tree. 6. Calculating the module eigengenes (MEs) for each module. The Pearson test was then used to determine the correlation between MEs and macrophages. A significant connection between the module and macrophages was found when P<0.05. By doing so, we identified a set of functionally similar genes that are associated with macrophage proportions. Subsequently, we intersected the genes in the modules most correlated with macrophages in the above three datasets with the macrophage marker genes in the single-cell dataset and displayed them as Venn diagrams.

### Consensus clustering analysis

Using the R package “ConsensusClusterPlus”, unsupervised hierarchical clustering was implemented to determine macrophage-related molecular subtypes in the TCGA-PRAD cohort according to the expression of macrophage-related intersection genes. The prognostic differences between the two molecular subtypes were compared using Kaplan-Meier (K-M) analysis. Dissimilarities in clinicopathologic traits between the two subtypes were compared utilizing the chi-square test and demonstrated by the heat map. For comparing dissimilarities in biological pathways between the two subtypes, gene set variation analysis (GSVA) was implemented with the “GSVA” R package.

### Immune microenvironment analysis

The single-sample gene set enrichment analysis (ssGSEA) was implemented to compute immune cell infiltration and immune-related pathway activity scores for each sample. The two-sample Wilcoxon test was performed to compare the differences in these immunization characteristics between different groups. Meanwhile, the immune cell infiltration abundance in PCa patients from the TCGA-PRAD cohort was measured using seven different software programs. The distinctions in abundance between different subtypes were compared, and Pearson’s correlation between immune cell content and risk scores was calculated. Additionally, we compared the gene expression of common human leukocyte antigen (HLA) and immune checkpoints in different groups. The TME difference between different groups was investigated with the R package “ESTIMATE” based on the ssGSEA result. In addition, the immune subtype profiles of the TGGA-PRAD cohort were obtained from the UCSC-Xena public data hub. Then, based on macrophage-related molecular subtypes, the distinction in immune subtypes between risk groups was compared via the “RColorBrewer” R package.

### Immunotherapy response

The immunophenoscore (IPS) of PCa samples obtained from The Cancer Immunome Atlas (TCIA, https://tcia.at/) was utilized to predict the immunotherapeutic response [37]. Immune reactivity is higher with a higher IPS score. The possible response to immune checkpoint blockade in PCa was predicted with the tumor immune dysfunction and exclusion (TIDE). Immunotherapy responses are better when the TIDE score is lower.

### Development and validation of the prognostic signature

To begin with, we used the “DESeq2” R package to determine the differentially expressed genes (DEGs) between the macrophage-related molecular subtypes with the threshold set to “P-value<0.05 and |log2FoldChange|> 1”. To identify prognostically relevant DEGs, we implemented a univariate Cox regression analysis. Next, we used the TCGA-PRAD cohort containing 497 prostate cancer patients as a training set. For a further selection of the most predictive DEGs, the least absolute shrinkage and selection operator (LASSO) Cox regression analysis with 10-fold cross-validation was performed in the TCGA training cohort with the R package “glmnet”. Based on the candidate DEGs obtained from the above screening, a macrophage-related prognosis signature was developed using forward stepwise selection and multivariate Cox regression. Utilizing the median risk score value, the patients from the TCGA cohort were classified into the low-risk or high-risk groups. With the “survivalROC” package, the area under the curve (AUC) of the receiver operating characteristic (ROC) curve was computed to verify the signature’s ability to predict prognosis. Using the R package “survminer”, the Kaplan–Meier (KM) method was utilized to implement the survival analysis, and the log-rank test was performed to determine the statistical significance of the differences. The robustness of the signature was also validated through KM analysis and AUC in eight completely independent datasets. Furthermore, univariate and multivariate Cox regression analyses confirmed that MRRS was an independent prognostic factor for PCa, and a clinically applicable nomogram was developed.

### Pathway and function enrichment analysis

Three different aspects of gene function were examined using Gene Ontology (GO) enrichment analysis, including biological processes, molecular functions, and cellular components. Through the Kyoto Encyclopedia of Genes and Genomes (KEGG) enrichment analysis, certain genes were searched to find their biological pathways. Based on DEGs between macrophage-related clusters, we conducted GO and KEGG analyses via the R package “clusterProfiler”. Gene set enrichment analysis (GSEA) was utilized to examine the differences in function and associated pathways between high- and low-risk samples using the R package “clusterProfiler”.

### Identifying the most important gene in the signature

Generally, if two gene products have similar functions, they will have high semantic similarity and have close gene ontology term trees [38]. Based on the “mgeneSim” function, which measures similarity by computing the geometric mean of molecular functions and cellular components, we assessed the significance of each gene to other genes in the signature by calculating the average similarity [39]. Additionally, we explored the hub gene expression in cells in the GSE141445 dataset and six PCa single-cell datasets in the tumor immune single-cell hub (TISCH) database. Moreover, the Tumor Immune Estimation Resource 2.0 (TIMER2.0) database (http://timer.cistrome.org/) evaluated the association between NCF4 expression and immune cell infiltration in PCa. Pairwise difference analysis was implemented to compare NCF4 expression differences in prostate tumor tissues and normal tissues. Utilizing the Human Protein Atlas (HPA) database, we verified whether NCF4 expression in PCa was different at the protein level from that in normal prostate tissue. Finally, we also explored the differences in clinicopathologic features and prognosis between the NCF4 high- and low-expression groups.

### Statistical analysis

Data processing, statistical analysis, and plotting were all conducted with R software (version 4.2.0). For the comparison of non-normally distributed continuous variables between two groups, the Wilcoxon rank-sum was applied. For the comparison of categorical variables between two groups, the chi-square test was utilized. Unless otherwise stated, the threshold for statistically significant differences was set to p-value <0.05.

### Data availability statement

The data used in this study are openly available in the TCGA database (https://portal.gdc.cancer.gov/, (accessed on 7 August 2022)), cBioPortal for Cancer Genomics (https://www.cbioportal.org/ (accessed on 11 August 2022)), CPGEA database (http://www.cpgea.com/download.php, (accessed on 13 August 2022)), and GEO database (https://www.ncbi.nlm.nih.gov/geo/, (accessed on 14 August 2022)).

## RESULTS

### Macrophage marker genes identification

**Figure 1:**
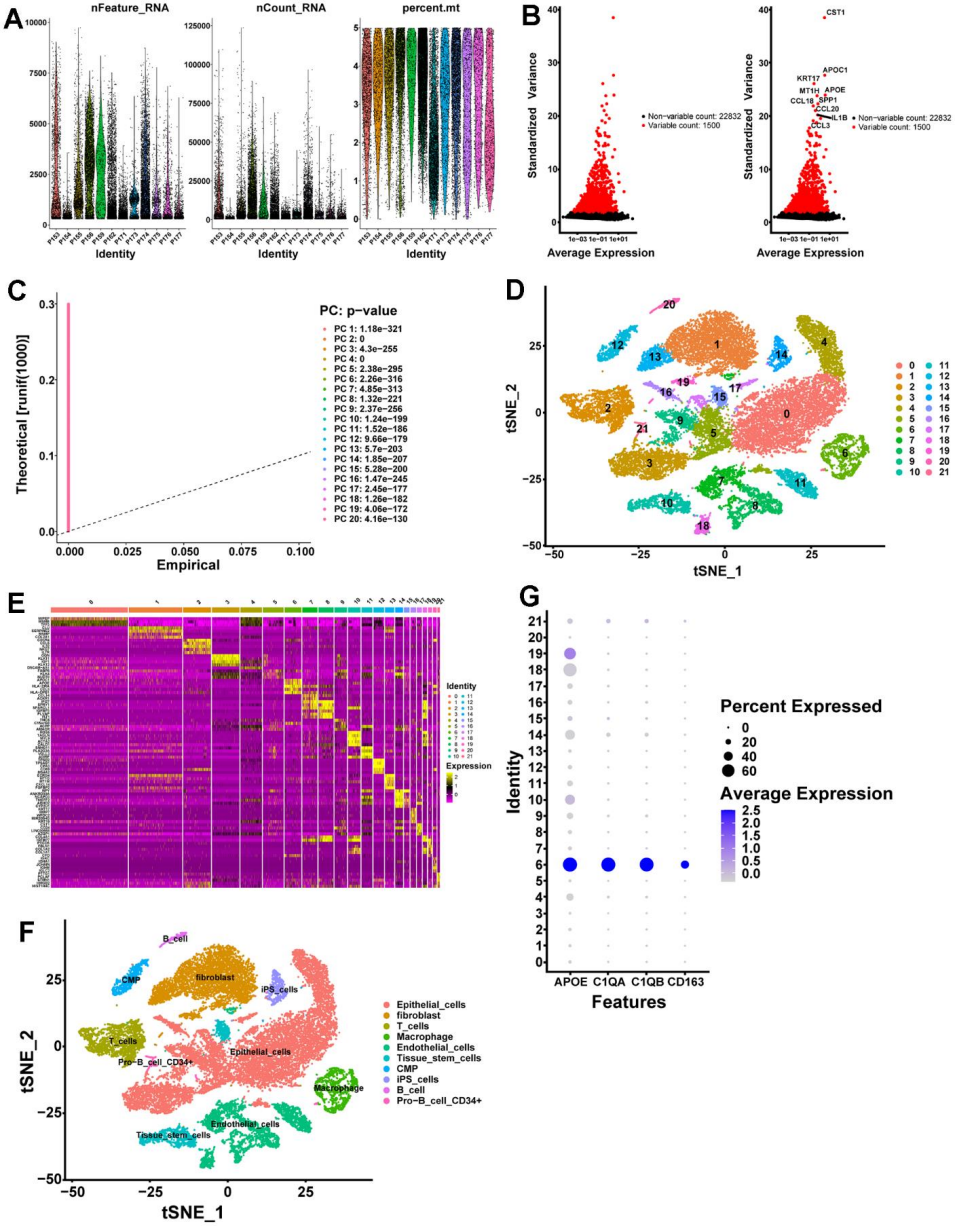
**scRNA-seq analysis identifies marker genes for macrophages.** (**A**) A total of 25999 eligible cells were identified after quality control of scRNA-seq data with twelve PCa samples. (**B**) Variation in gene expression across all PCa cells is shown in the variance plot. The black dots represent non-variable genes, while the red dots represent highly variable genes. (**C**) A P-value of 0.05 identified 20 PCs. (**D**) A t-SNE algorithm was applied to visualize 22 clusters. (**E**) The top 5 marker genes in each cell cluster are displayed in a heatmap. Genes with high expression are yellow, and genes with low expression are purple. (**F**) Cell types identified by marker genes. (**G**) Macrophage marker gene expression levels in each cell cluster are represented by bubble plots.

The scRNA-seq data of GSE141445 contain 102899 cells from 12 primary PCa samples. Following strict quality control, 25999 cells were retained for further analysis (Figure 1A). Based on the normalized data, the top 1500 highly variable genes were selected (Figure 1B). Utilizing the top 1500 variable genes, PCA was implemented to reduce the dimensionality, and 20 principal components (PCs) were selected with a p-value < 0.05 for subsequent analysis (Figure 1C). Following that, 22 independent cell clusters were identified utilizing the t-SNE algorithm (Figure 1D). Across the 22 clusters, we identified 4054 differentially expressed marker genes shown in Supplementary Table 2. The heatmap displaying the relative expression of the top 5 marker genes within each cluster is shown in Figure 1E. Next, the cell identity of each cluster was annotated and cells in cluster 6 were designated as macrophages (Figure 1F and Table 1). Meanwhile, the expression of macrophage marker genes for PCa from the CellMarker database was visualized by bubble plots (Figure 1G), which further identified cluster 6 cells as macrophages. Ultimately, we identified 320 TAMs marker genes for PCa in this study (Supplementary Table 3).

**Table 1:** Clusters annotate.

**Seurat clusters**	**Cell type**
0	Epithelial_cells
1	fibroblast
2	T_cells
3	Epithelial_cells
4	Epithelial_cells
5	Epithelial_cells
6	Macrophage
7	Endothelial_cells
8	Endothelial_cells
9	Epithelial_cells
10	Tissue_stem_cells
11	Endothelial_cells
12	CMP
13	fibroblast
14	iPS_cells
15	Tissue_stem_cells
16	Epithelial_cells
17	Epithelial_cells
18	Endothelial_cells
19	fibroblast
20	B_cell
21	Pro-B_cell_CD34+

### Cell communication analysis between cluster C6 and other clusters

**Figure 2:**
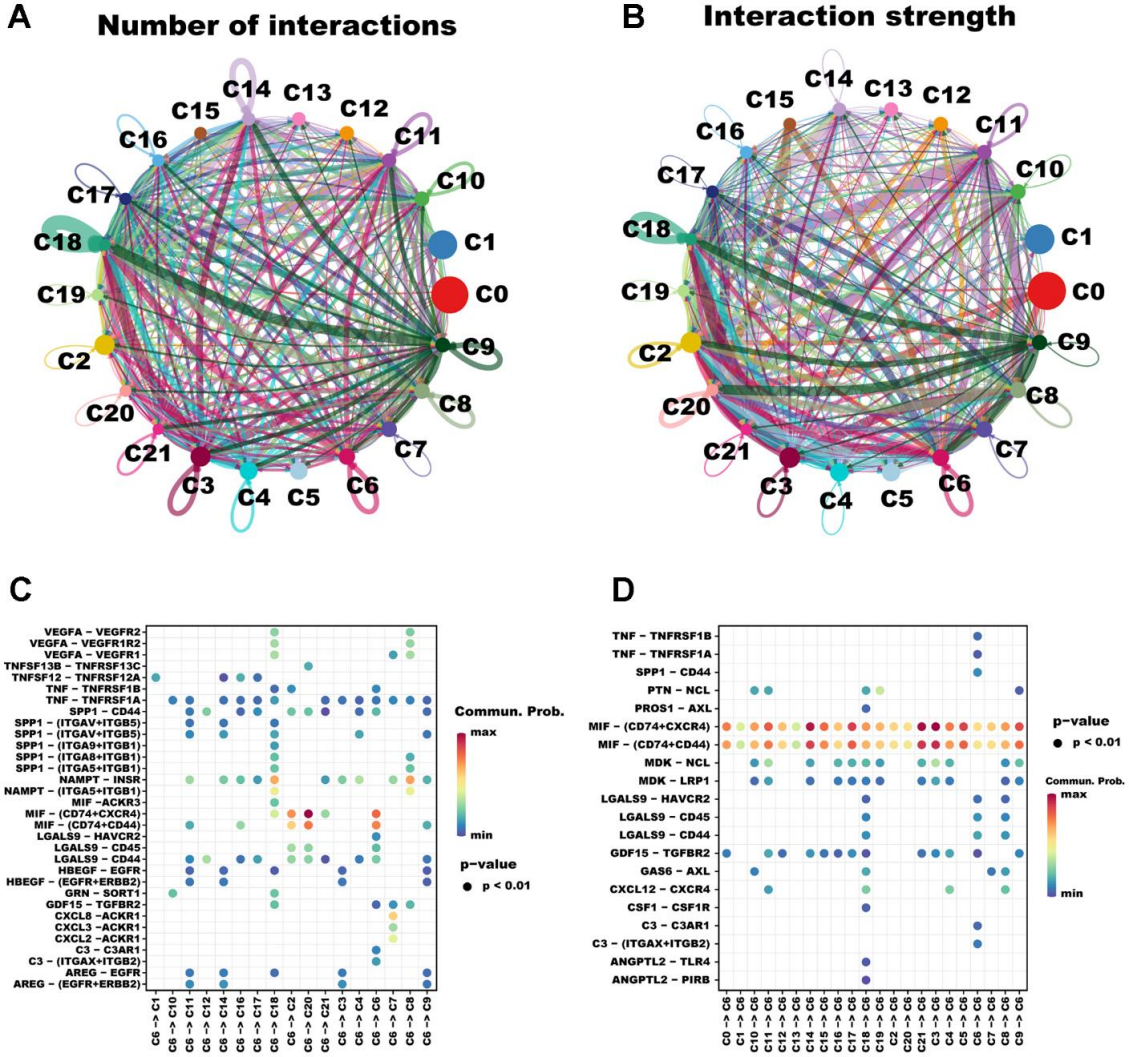
**The cellular communication between C6 and other clusters.** The circle diagrams show the high intercellular interactions concerning the number (**A**) and strength (**B**) of ligand–receptor interactions among the 22 clusters. Interactions between overexpressed ligands and receptors are shown in the bubble chart when C6 cluster cells act as the ligand (**C**) and receptor (**D**) cells, respectively. Permutation test P-values are represented by bubble size, while interaction possibilities are represented by color.

In multicellular organisms, the basic processes of cellular activity are dependent on intercellular interactions, and ligand-receptor pairs are the primary mechanisms of intercellular communication. Considering that TAMs play a pivotal role in tumor progression, we analyzed cell communication between cluster C6 and other clusters using Cell Chat. A high degree of intercellular correlation was found within 22 clusters based on the number and strength of ligand-receptor interactions (Figure 2A, 2B). According to the ligand-receptor information for each cluster, the C6 cluster affects other clusters via ligand-receptor pairs; for instance, the C6 cluster affects C2 and C20 clusters mainly through MIF - (CD74+CXCR4) and MIF - (CD74+CD44) (Figure 2C). Notably, other cell clusters including the C6 cluster itself also affect the C6 cluster mainly through MIF - (CD74+CXCR4) and MIF - (CD74+CD44) (Figure 2D). Taken together, intercellular communication provides new ideas for the development of novel therapies targeting macrophages.

### Identification of genes related to macrophage infiltration in PCa through WGCNA

**Figure 3:**
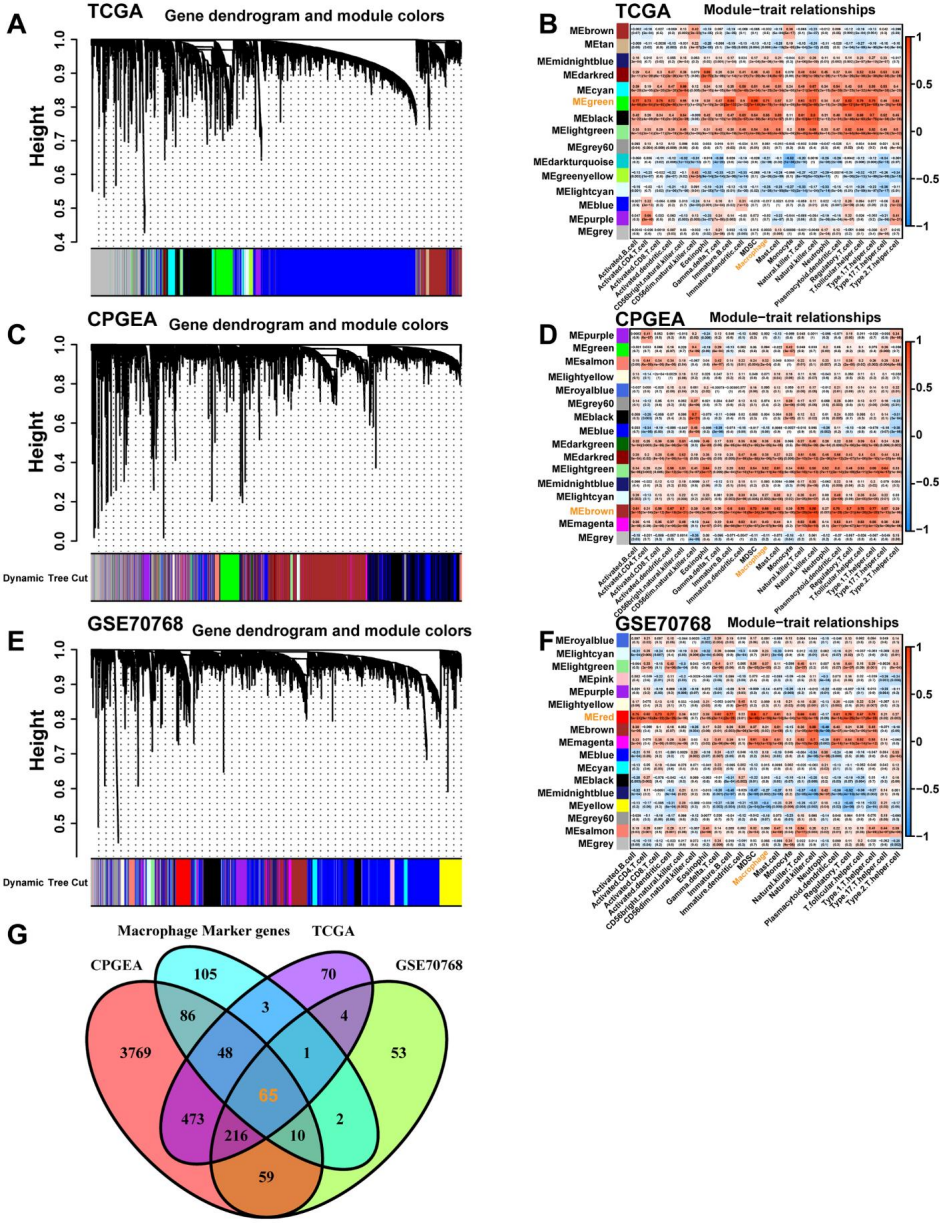
The co-expression network and heatmap illustrating the association between module eigengenes and macrophages were constructed through WGCNA based on the bulk RNA-seq data from the TCGA-PRAD database (**A**, **B**), the CPGEA dataset (**C**, **D**), and the GSE70768 database (**E**, **F**), respectively. (**G**) The Venn diagram displays the intersection of genes between macrophage-related genes selected from the above different datasets and macrophage marker genes from the scRNA-seq data.

In the TCGA-PRAD cohort dataset, genes were classified into fifteen modules (Figure 3A), in which the green module, containing 880 genes (Supplementary Table 4), was highly correlated with macrophage (Figure 3B, R2 = 0.71, P = 6e−78). In the CPGEA cohort dataset, genes were divided into sixteen modules (Figure 3C), in which the brown module, containing 4726 genes (Supplementary Table 5), was highly correlated with macrophage (Figure 3D, R2 = 0.66, P = 2e−18). In the GSE70768 cohort dataset, genes were divided into seventeen modules (Figure 3E), in which the red module, containing 410 genes (Supplementary Table 6), was highly correlated with macrophage (Figure 3F, R2 = 0.70, P = 1e−19). Next, we intersected these genes in the highly macrophage-relevant modules of the three datasets described above with the macrophage marker genes in the single-cell dataset (Figure 3G) and finally obtained 65 genes highly correlated with macrophages for subsequent analysis (Supplementary Table 7).

### Identification of macrophage-related molecular subtypes in PCa and characterization of the immune landscape between subtypes

**Figure 4:**
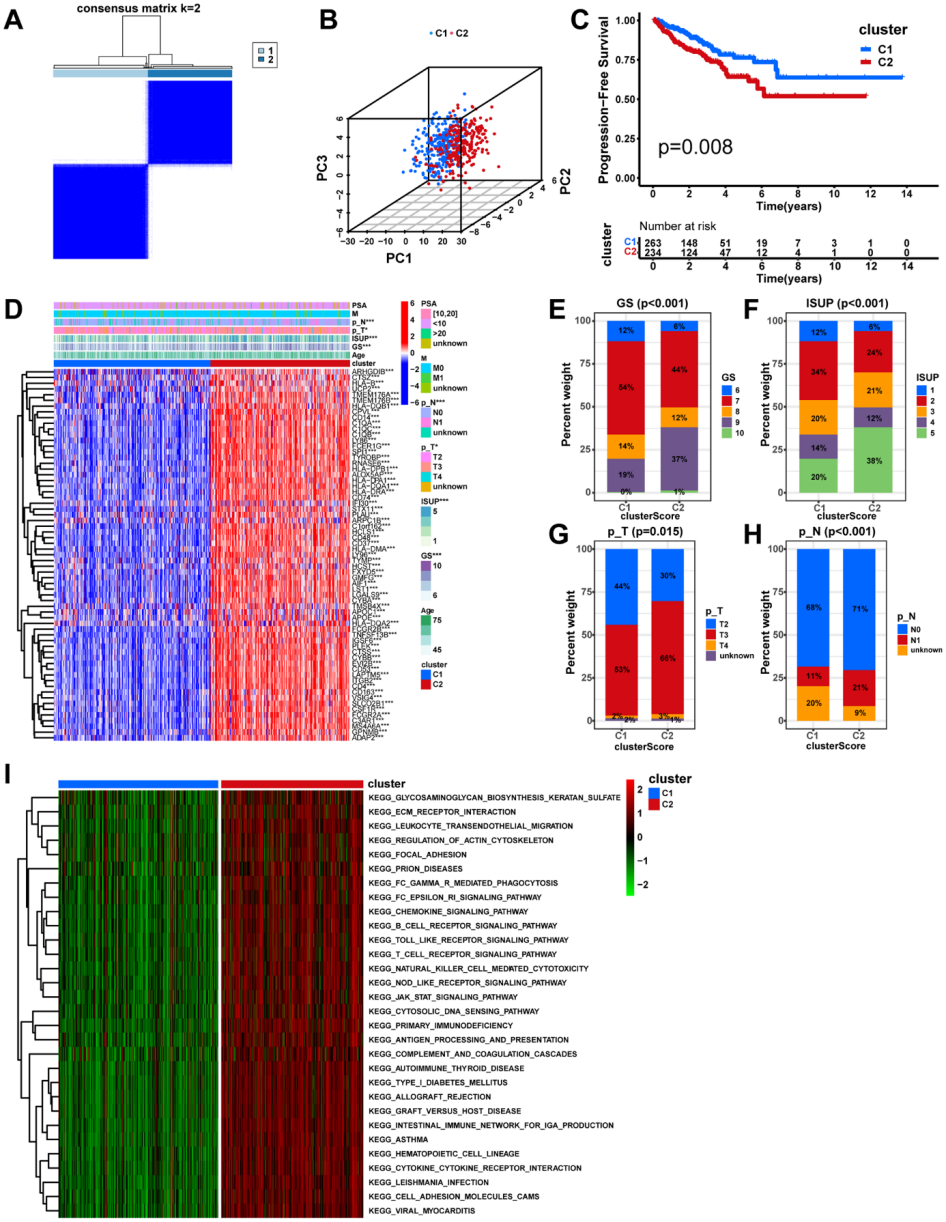
**Unsupervised consensus analysis in the TCGA cohort.** (**A**) Based on the expression of the 65 macrophage-related intersection genes, PCa patients in the TCGA cohort were separated into two distinct clusters when k = 2. (**B**) According to the 3D PCA plots, the cluster well-differentiated PCa patients from one another. (**C**) The KM analysis between different clusters. (**D**) Heatmap showing the expression levels of the 65 macrophage-related intersection genes and the distribution of clinicopathological features between clusters. The fractions of GS (**E**) ISUP (**F**), pathologic T stage (**G**), and pathologic N stage (**H**) between cluster groups. (**I**) The heatmap displays the GSVA result between distinct macrophage-related clusters. ns, not significant; **P < 0.01; ***P < 0.001.

To further explore inter-tumor macrophage heterogeneity, the expression of the 65 macrophage-related intersection genes described above was analyzed using unsupervised consensus analysis to categorize 497 PCa patients in the TCGA-PRAD cohort into two distinct subtypes, with 263 samples in macrophage-related cluster 1 and 234 samples in macrophage-related cluster 2 (Figure 4A, Supplementary Figure 1). According to 3D PCA, 65 macrophage-related intersecting genes could well distinguish the two clusters (Figure 4B). The KM analysis showed that cluster C2 had a worse prognosis (Figure 3C, p=0.008). The heatmap showed that all 65 macrophage-related intersecting genes were significantly highly expressed in cluster 2, and there were significant distributional differences of GS, ISUP, pathological T-stage, and pathological N-stage between the two molecular subtypes (Figure 3D). Of these, cluster 2 had significantly higher proportions of high-level GS (Figure 4E, p<0.001), ISUP (Figure 4F, p<0.001), pathologic T-stage (Figure 4G, p=0.015), and pathologic N-stage (Figure 4H, p<0.001). In summary, the results of this study imply that 65 macrophage-related intersecting genes can be used to distinguish two subtypes of PCa, each of which has an entirely different prognosis and clinicopathologic profile.

**Figure 5:**
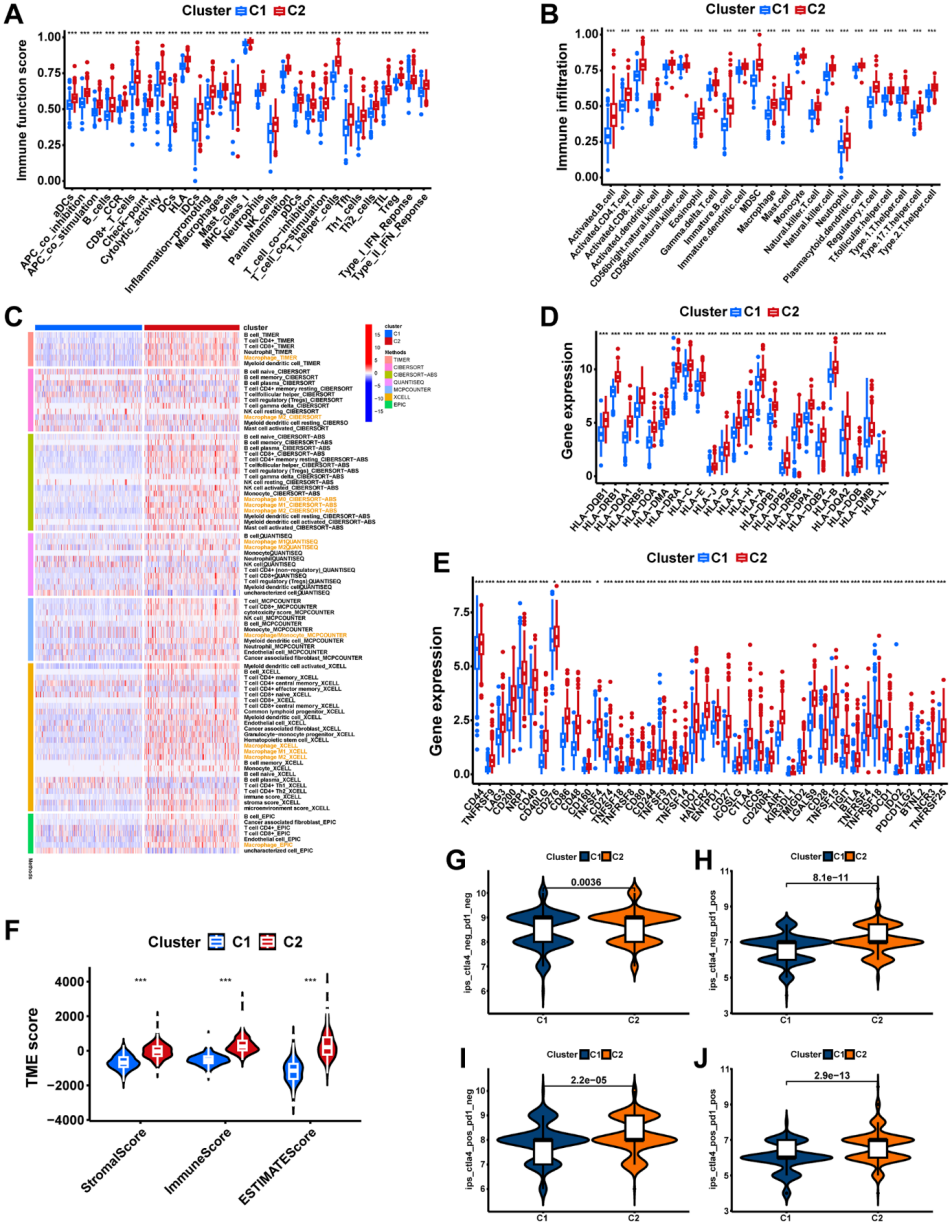
**The immune landscape of macrophage-related molecular subtypes.** (**A**) The boxplot displays the difference in immune-related functions between different clusters. (**B**) Two clusters have different levels of immune cell infiltration. (**C**) Seven immune infiltration software displays the immune infiltration between different clusters. (**D**) Comparing the expression of HLA molecules between two clusters. (**E**) Expression of immune checkpoint molecules between the two clusters. (**F**) The comparisons of stromal score, immune score, and estimate score between clusters. Comparing immunophenoscores (IPS) across clusters; (**G**) CTLA4−_PD1−, (**H**) CTLA4−_PD1+, (**I**) CTLA4+_PD1−, and (**J**) CTLA4+_PD1+. ns, not significant; **P < 0.01; ***P < 0.001.

The GSVA was implemented to identify the potential mechanisms that may explain the differences between the two subtypes. Notably, virtually all pathways associated with tumor progression, intercellular communication, and immunity, including the JAK-STAT signaling pathway, ECM-receptor interaction, Chemokine signaling pathway, and T cell receptor signaling pathway, were significantly enriched in cluster 2 (Figure 4I, p<0.05), which may account for the poorer prognosis. To further explore the intrinsic reasons for the differences between the two subtypes, a comparison was made between the two subtypes in terms of the TIME and the activities of immune-related pathways. Notably, all immune functions including the checkpoint, HLA, and macrophages were more active in cluster 2 (Figure 5A, p<0.05). In comparison with cluster 1, cluster 2 demonstrated significantly higher infiltration abundance of all immune cells, including macrophages, as determined by ssGSEA (Figure 5B, p<0.05). In addition, the seven immune infiltration algorithms were consistent, with more immune cell infiltration including macrophage, macrophage M1, and macrophage M2 in cluster 2 (Figure 5C). Moreover, we compared gene expression between different clusters for immune checkpoints and HLAs. All common HLA genes were significantly highly expressed in cluster 2 (Figure 5D, p<0.05). Immune checkpoint genes, including B7H3 (CD276), HAVCR2, CTLA4, TIGIT, and PD-1 (PDCD1), were highly expressed in cluster 2 (Figure 5E, p<0.05). Using the ESTIMATE algorithm, we calculated the stromal, immune, and estimate scores based on the gene expression profiles of each PCa sample, and the results indicated that all these scores were significantly higher in cluster 2 (Figure 5F, p<0.001). Lastly, IPS files downloaded from the TCIA database were utilized to analyze the response to immunotherapy in patients with PCa to determine whether macrophage-related clusters can predict ICI responses. The IPS, the IPS-CTLA4 score, the IPS-PD1 blocker score, and the IPS-CTLA4+PD1 blocker score were significantly higher in cluster 2 than in cluster 1 (Figure 5G–5J, p<0.01), which demonstrated a more immunogenic phenotype in cluster 2, suggesting that immunotherapy might be more beneficial to PCa patients in cluster 2. In conclusion, macrophage-associated subtypes have significantly different pathway activation status, tumor immune microenvironment, and immunotherapeutic efficacy, which is of great significance for clinical practice and research in PCa.

### Development and validation of a macrophage-related signature (MRS)

**Figure 6:**
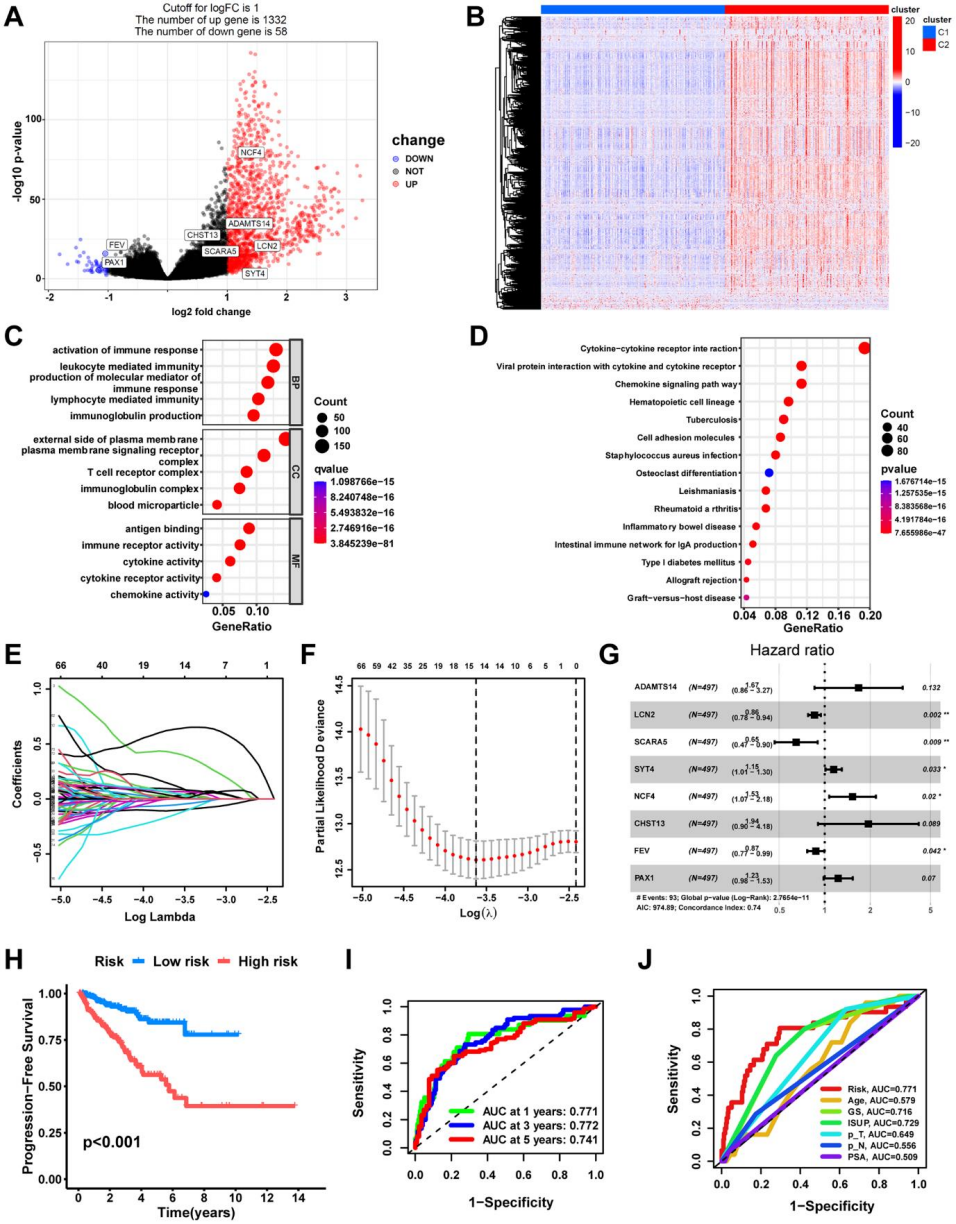
**Development of a macrophage-related signature (MRS).** (**A**) Volcano plot of DEGs between macrophage-related molecular subtypes in TCGA cohort. Significant DEGs were found when P < 0.05 and |log2FoldChange|> 1. Genes that are upregulated are shown in red, and genes that are downregulated are shown in blue. (**B**) Heatmap of DEGs. Bubble plots of the GO (**C**) and KEGG pathways (**D**) functional enrichment of DEGs. (**E**) Based on macrophage-related clusters, LASSO Cox regression analysis selected eight genes. (**F**) Cross-validation for tuning parameter selection in the LASSO model. (**G**) Forest plot of multivariate Cox regression result. (**H**) The Kaplan-Meier curves in the training cohort (TCGA). (**I**) The AUC at 1-, 3-, and 5-years of prognostic models in the training cohort. (**J**) ROC curves evaluate the predictive accuracy of the risk score and clinicopathological features. ns, not significant; **P < 0.01; ***P < 0.001.

First, a total of 1,390 significant DEGs, including 1,332 up-regulated genes and 58 down-regulated genes, were obtained by differential expression analysis between the macrophage-related molecular subtypes (Figure 6A, 6B). The GO analysis indicated that DEGs were primarily enriched in functions related to immunity and intercellular communication, including the activation of immune response, plasma membrane signaling receptor, T cell receptor complex, antigen binding, and immune receptor activity (Figure 6C). The KEGG enrichment results showed that the Cytokine−cytokine receptor interaction, Chemokine signaling pathway, and Cell adhesion molecules were the enriched pathways for DEGs (Figure 6D).

The univariate Cox regression analysis screened out 171 DEGs related to progress free survival (PFS) in PCa (Supplementary Table 8). Next, 14 DEGs were screened out as optimal prognostic biomarkers by LASSO regression analysis with 10-fold cross-validation using the TCGA cohort with 497 PCa patients as a training set (Figure 6E, 6F). Following that, the model with the lowest Akaike information criterion (AIC) value was generated via multivariate Cox regression analysis. Finally, a macrophage-related signature consisting of eight DEGs, including ADAMTS14, LCN2, SCARA5, SYT4, NCF4, CHST13, FEV, and PAX1, was constructed (Figure 6G, Supplementary Table 9). The risk score was computed utilizing the coefficient of each gene in the signature: MRS = (0.5156*ADAMTS14 expression) + (-0.1546*LCN2expression) + (-0.4346*SCARA5expression) + (0.1359*SYT4expression) + (0.4235*NCF4expression) + (0.6643*CHST13expression) + (-0.1348*FEVexpression) + (0.2045*PAX1expression).

**Figure 7:**
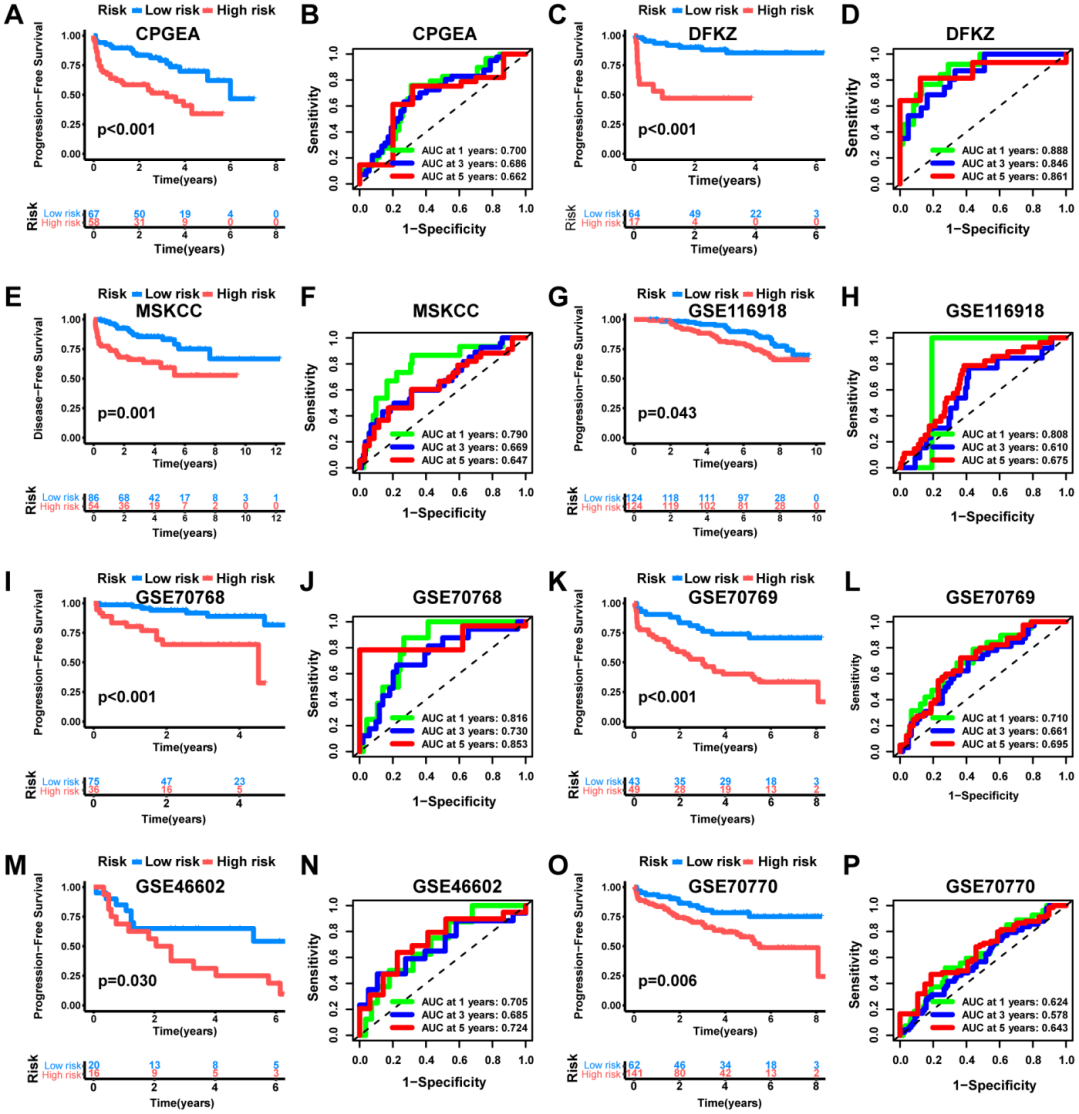
**External validation of the MRS.** KM analysis as well as ROC curve and AUC of MRS in CPGEA cohort (**A**, **B**), DFKZ cohort (**C**, **D**), MSKCC cohort (**E**, **F**), GSE116918 cohort (**G**, **H**), GSE70768 cohort (**I**, **J**), GSE70769 cohort (**K**, **L**), GSE46602 cohort (**M**, **N**) and GSE70770 cohort (**O**, **P**).

Subsequently, we evaluated the prognostic signature’s predictive value. According to the median risk score value, PCa patients in the training set were divided into high-risk and low-risk groups. Compared with the low-risk group, the high-risk group possessed significantly poorer PFS (Figure 6H, p<0.001). The AUC of 1-, 3-, and 5-year in the training cohort were 0.771, 0.772, and 0.741, respectively (Figure 6I). The ROC curves illustrated that the risk score was notably superior to other clinicopathological variables in predicting the PFS of PCa patients (Figure 6J). Additionally, eight independent datasets (CPGEA, DFKZ, MSKCC, GSE116918, GSE70768, GSE70769, GSE46602, and GSE70770) were validated externally to further verify the signature’s robustness. Consistently, all cohorts demonstrated significantly shorter PFS time for patients in the high-risk group, containing the CPGEA cohort (n=125, p<0.001, Figure 7A), the DFKZ cohort (n=81, p<0.001, Figure 7C), the MSKCC cohort (n=140, p=0.001, Figure 7E), the GSE116918 cohort (n=248, p=0.043, Figure 7G), the GSE70768 cohort (n=111, p<0.001, Figure 7I), the GSE70769 cohort (n=92, p<0.001, Figure 7K), the GSE46602 cohort (n=36, p=0.030, Figure 7M), and the GSE70770 cohort (n=203, p=0.006, Figure 7O). Moreover, the ROC curves confirmed that the signature held good predictive value in these datasets (Figure 7). Overall, this signature is robust and has promising application prospects.

### Independent prognostic analysis and nomogram establishment

**Figure 8:**
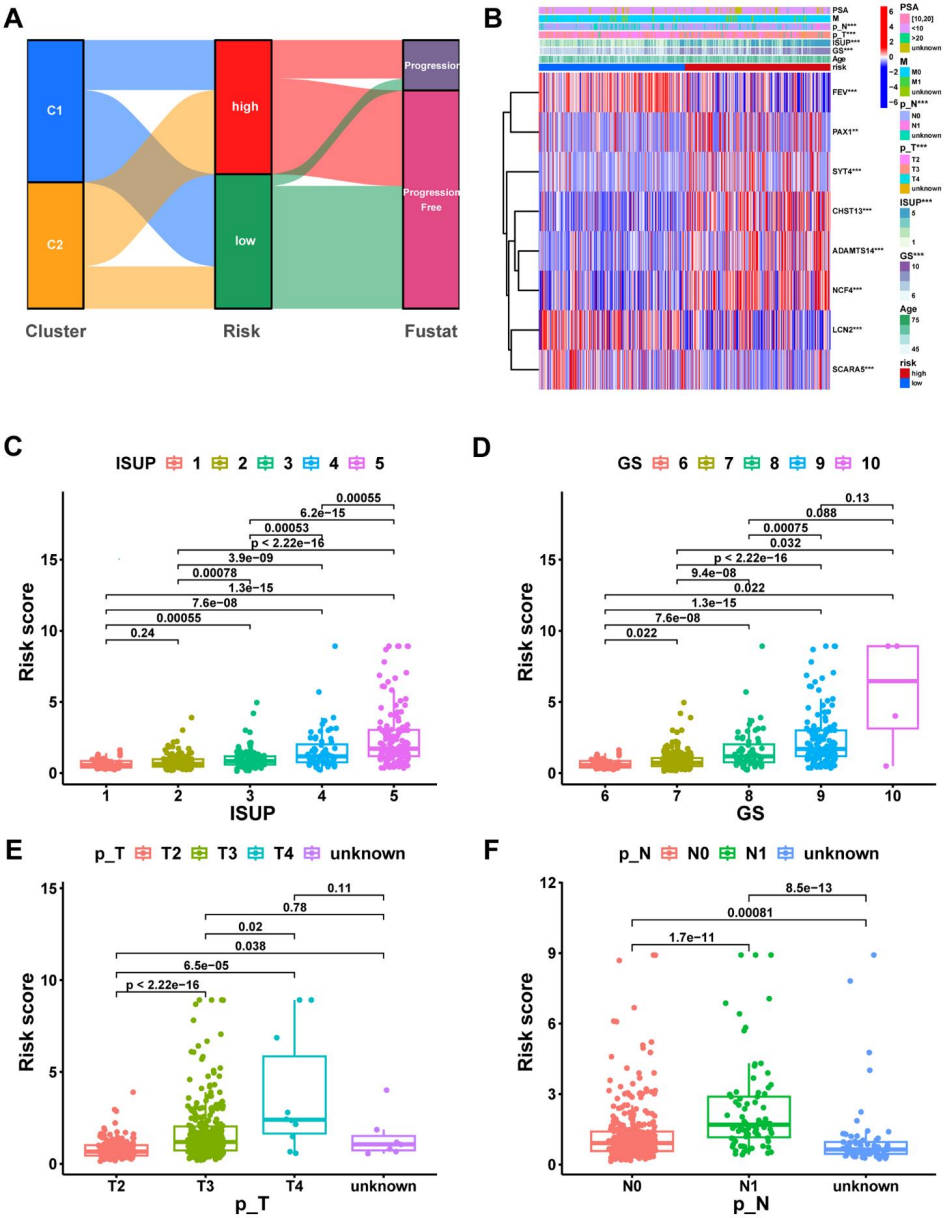
**Correlation analysis of risk scores with clinical characteristics.** (**A**) The potential correlation between clusters, risk score, and survival status was displayed by the Sankey diagram. (**B**) Heatmap of the MRS signature and clinicopathological characteristics. Boxplot showing the correlation between risk score and different ISUP stages (**C**), GS stages (**D**), pathologic T stages (**E**), and pathologic N stages (**F**). ns, not significant; **P < 0.01; ***P < 0.001.

**Figure 9:**
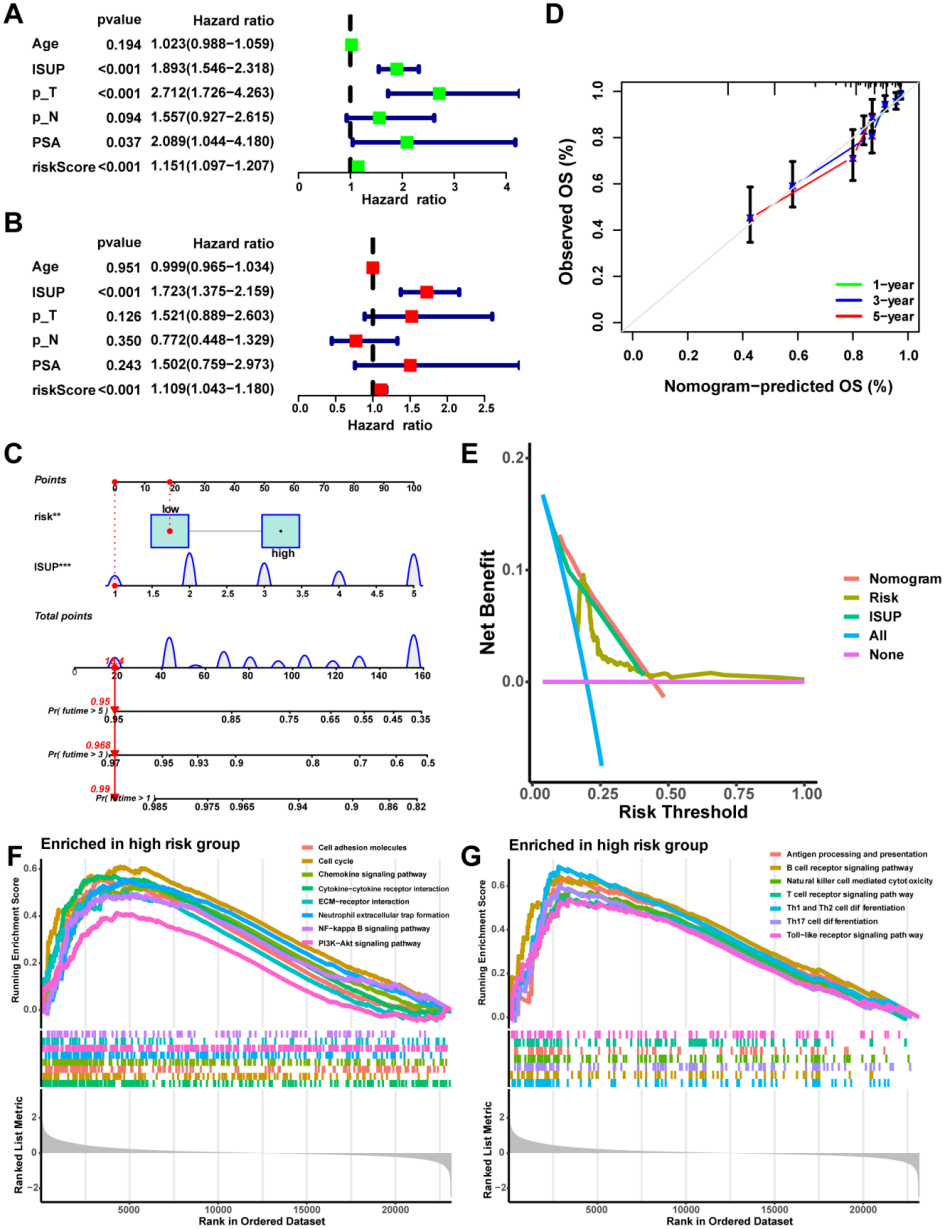
**Establishment and assessment of the nomogram.** (**A**) Univariate Cox analysis of risk scores and clinical characteristics. (**B**) Multifactorial Cox analysis of risk scores and clinical characteristics. (**C**) Construction of the nomogram. (**D**) The calibration curve of the nomogram. (**E**) DCA diagram. Enrichment of tumor-related pathways (**F**) and immune-related pathways (**G**) in the high-risk group.

The Sankey diagram illustrated a relationship among macrophage-related subtypes, risk scores, and prognosis, in which patients with disease progression predominantly from the high-risk group (Figure 8A). In the TCGA-PRAD cohort, a significant correlation was found between risk scores and GS, ISUP, pathologic T-stage, and pathologic N-stage, with higher grades of these characteristics indicating a significant increase in risk scores (Figure 8B–8F, P < 0.05). Moreover, both univariate and multivariate Cox regression analyses confirmed that MRS was an independent prognostic factor for PCa (Figure 9A, 9B). Consequently, we constructed a clinically adapted nomogram to predict the 1-, 3-, and 5-year prognosis for PCa patients depending on the results of Cox regression analysis (Figure 9C). Nomogram predictions and observed probabilities showed excellent concordance on the calibration plot (Figure 9D). Additionally, the decision curve analysis (DCA) suggested that the MRS provided a greater net clinical benefit than other clinicopathological features (Figure 9E). In summary, the above findings suggest that MRS is an independent prognostic factor for PCa and the MRS-based nomogram has good prognostic predictive value for PCa patients.

### GSEA between different risk groups

To provide further insight into the underlying mechanisms by which high-risk subgroups have worse pathologic stages and prognosis, we performed GSEA to identify the KEGG pathways most significantly enriched in high-risk groups. The results indicate that several classical intercellular communication-related pathways, containing the Chemokine signaling pathway, Cytokine−cytokine receptor interaction, and ECM−receptor interaction, as well as tumor-related pathways, containing NF−kappa B signaling pathway, PI3K−Akt signaling pathway, and Cell cycle, were enriched in the high-risk group (Figure 9F). Additionally, many immune-related pathways were also enriched in the high-risk group, including Antigen processing and presentation, B cell receptor signaling pathway, T cell receptor signaling pathway, and Natural killer cell mediated cytotoxicity (Figure 9G). This partly explains the significantly higher pathologic grade and significantly worse prognosis in the high-risk group.

### The immune landscape of the signature

**Figure 10:**
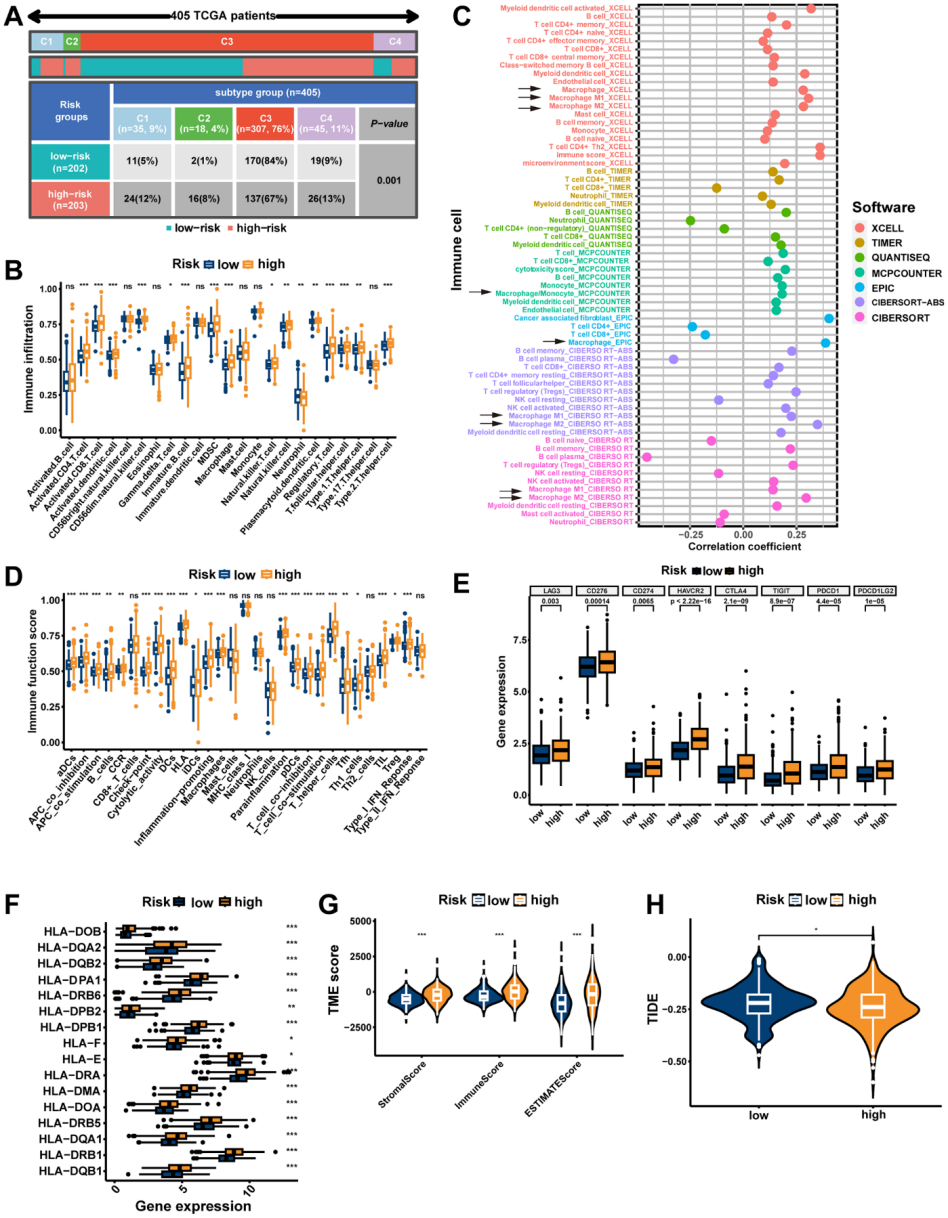
**The immune landscape of the signature.** (**A**) Different risk groups have different immune subtypes. (**B**) Differences between the two risk groups in immune cell infiltration. (**C**) The bubble plot shows the correlation between different immune cells and risk scores. (**D**) The comparison of immune-related functions or pathways between the two risk groups. The comparison of immune checkpoint (**E**) and HLA (**F**) molecules expression between the two risk groups. (**G**) Stromal score, immune score, and estimate score between the two risk groups. (**H**) Comparison of the tumor immune dysfunction and exclusion (TIDE) prediction scores in the low- and high-risk groups. ns, not significant; **P < 0.01; ***P < 0.001.

Significant differences in immunophenotypes between risk groups were found by comparing PCa immune subtype proportions in different risk groups, with significantly higher proportions of C1 (Wound Healing) subtype, C2 (IFN-γ Dominant) subtype, and C4 (Lymphocyte Depleted) subtype and significantly lower proportions of C3 (Inflammatory) subtype in the high-risk group (Figure 10A, p=0.001). Notably, it was shown that C1 subtype had elevated angiogenic gene expression with a high tumor cell proliferation rate; C2 subtype had the highest degree of M1/M2 macrophage polarization, which promoted tumor cell proliferation; C4 subtype exhibited a more prominent macrophage profile, with suppressed Th1 and a high M2 response; while C3 subtype had elevated expression of Th17 and Th1 genes and a low tumor cell proliferation rate [40]. Based on ssGSEA results, the high-risk group had more infiltration of immune cells, including macrophages (Figure 10B, p<0.001). Moreover, the seven immune infiltration algorithms were consistent, with risk scores positively correlating with the cellular content of macrophage, macrophage M1, and macrophage M2 (Figure 10C). The high-risk group possessed more active immune functions, including checkpoints, HLA, and macrophages (Figure 10D, p<0.001). Furthermore, in the high-risk group, immune checkpoints (including CTLA4 and PDCD1/PD-1) as well as HLA were highly expressed (Figure 10E, 10F, p<0.05). Consistently, tumor microenvironment analyses showed that stromal scores, immune scores, and estimate scores were higher in the high-risk group (Figure 10G, p<0.001). Moreover, we examined whether MRS could predict immunotherapy response in PCa patients. As measured by the TIDE algorithm, the TIDE score for the high-risk group was significantly lower than that of the low-risk group (Figure 10H, p<0.05), suggesting that anti-PD1/CTALA4 therapy may be more beneficial for high-risk patients. In summary, possessing significant differences in immunophenotyping, TIME and immunotherapeutic response between the two risk groups suggests that MRS may contribute to research and clinical practice in PCa immunotherapy.

### Identification of the MRS core gene NCF4

**Figure 11:**
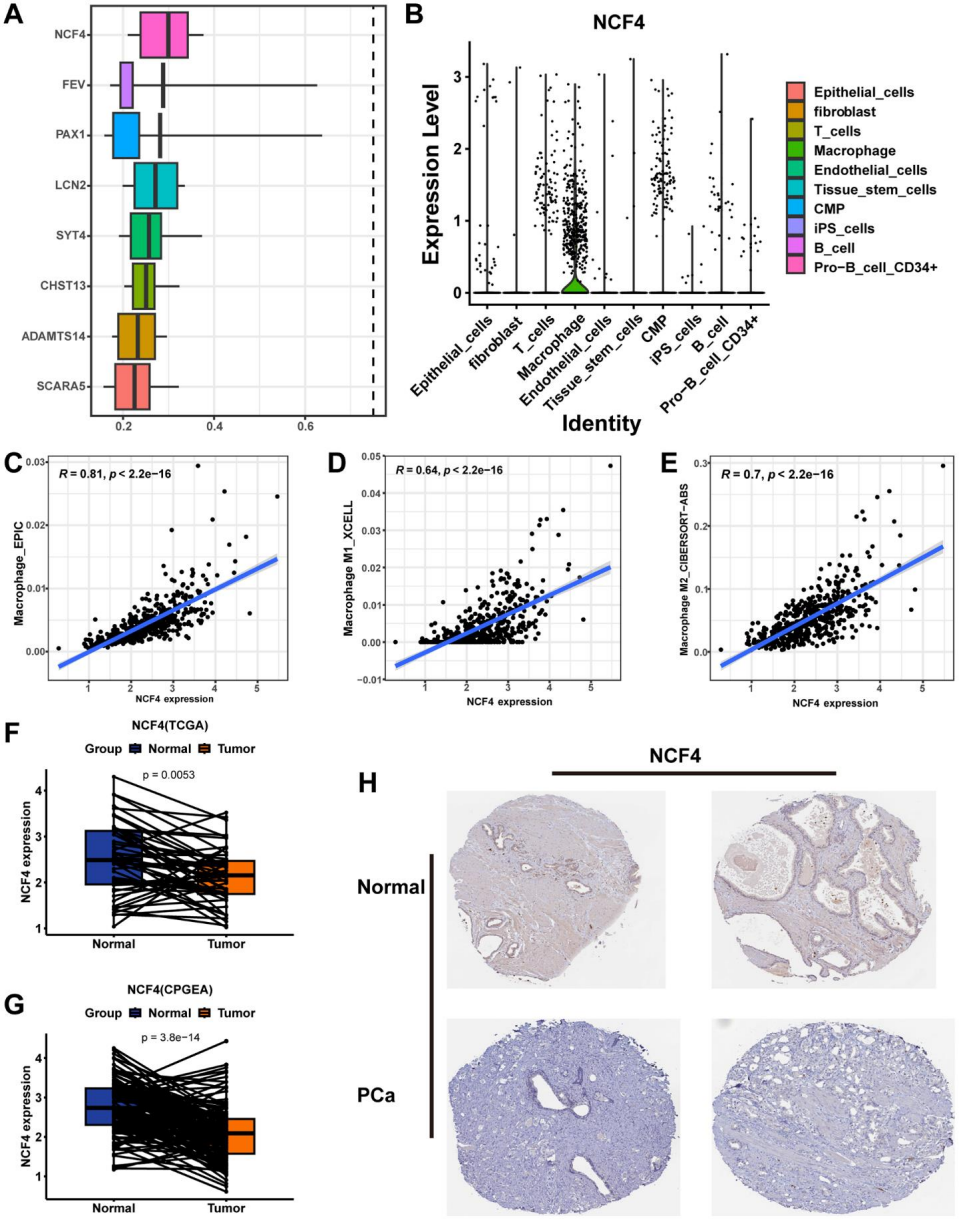
**Identifying NCF4 as the hub gene.** (**A**) The “mgeneSim” function reveals the hub gene NCF4 in the MRS. (**B**) The violin diagram shows the expression of NCF4 in various types of cells in GSE141445. Correlation scatter plots demonstrate the correlation of NCF4 expression levels with the content of macrophage cells (**C**), macrophage M1 cells (**D**), and macrophage M2 cells (**E**). Paired box plots demonstrating the difference in NCF4 expression between normal and cancerous prostate tissues in TCGA-PRAD cohort (**F**) and CPGEA cohort (**G**). (**H**) The immunohistochemical staining shows NCF4 expression at the protein level in normal and PCa tissues.

NCF4 was identified as a key gene in MRS using the “mgeneSim” function (Figure 11A). We then explored the expression of NCF4 in cell types in 12 primary prostate cancer samples from the single-cell dataset GSE141445. The results indicated that NCF4 was predominantly expressed in macrophages and rarely in other cells (Figure 11B). Additionally, all six PRAD single-cell datasets in the TISCH database were analyzed to determine the expression of NCF4 in immune and non-immune cells. Consistently, NCF4 was highly expressed predominantly in monocytes or macrophages and to a lesser extent in non-immune and tumor cells in the microenvironment (Supplementary Figure 2A). In TIMER 2.0, we further utilized multiple algorithms to examine the relationship between immune cell infiltration and NCF4 expression. The results indicated that macrophage, macrophage M1, and macrophage M2 were significantly positively correlated with NCF4 expression (Figure 11C–11E).

Additionally, we explored the expression of NCF4 in prostate tissues. Pairwise analysis revealed that NCF4 expression was significantly higher in normal tissues than in tumor tissues in the TCGA-PRAD cohort (Figure 11F, p=0.0053) and CPGEA cohort (Figure 11G, p<0.001). According to the HPA database immunohistochemical data, NCF4 was also expressed at lower protein levels in PCa than in normal tissues (Figure 11H). Furthermore, clinicopathologic analysis showed significant differences in clinicopathologic characteristics between patients with PCa in the high-expression and low-expression groups of NCF4 (Supplementary Figure 2B). Among them, there is a higher proportion of high-grade ISUP, high-grade GS, high-grade pathologic T-stage, high-grade pathologic N-stage, and higher age in the NCF4 high-expression group (Supplementary Figure 2C–2G). Meanwhile, patients in the NCF4 high expression group had a poorer prognosis (Supplementary Figure 2H). In summary, NCF4 is not only closely related to TAMs, but these results also inform the value of exploring NCF4 in future PCa studies.

## DISCUSSION

PCa is the most common tumor of the male genitourinary system and the second leading cause of cancer-related deaths in male patients [1]. High-grade PCa patients are at risk of developing resistance to treatment and progressing to advanced PCa, for which treatment options are limited. Among them, immunotherapy is one of the treatment methods for advanced tumors, but its efficacy in advanced PCa is not revolutionary. This is mainly due to PCa being referred to as a ‘cold tumor’ in its immunosuppressive microenvironment, where its immune system cannot be fully activated to fight against the tumor [41, 42]. However, recent studies have shown that the TME is dynamic, and a ‘cold tumor’ may have the potential to transform into a ‘hot tumor’ [43]. Considering that the majority of immune cells in the PCa TME are TAMs, targeting TAMs to activate anti-tumor immunity is a novel direction in PCa immunotherapy. Previous studies have shown that TAMs can induce PCa tumor cells to become resistant to chemotherapy, and castration-resistant PCa patients who are treated with docetaxel benefit from TAM inhibitors [44]. However, the role of TAMs in the TIME and immunotherapy of PCa is largely unexplored at present. Therefore, this study aimed to investigate the roles of TAMs-related genes in molecular stratification, prognosis, TME, and immunotherapeutic response in PCa.

TAMs, as an important component of the TIME, are now found to be used for molecular subtyping in various tumors in relevant studies. Su et al.’s study divided patients with hepatocellular carcinoma (HCC) into three subtypes (high, medium, and low expression types) based on the expression levels of TAMs-related genes, among which patients with high expression type had higher tumor grades, lower survival rates, and higher TIDE scores [45]. This suggests that HCC patients with high expression of TAMs-related genes have a worse prognosis and are also more prone to immune escape, demonstrating the potential of molecular subtyping in immunotherapy and prognosis prediction in HCC. Xie et al.’s study on TAMs molecular subtyping in non-small cell lung cancer also shows that patients with high TAMs-related gene expression type have immune suppression and tumor immune escape phenomena [46].

Immunotherapy, as an emerging treatment strategy for advanced PCa, its therapeutic agents mainly include Sipuleucel-T, an autologous vaccine targeting PAP, and ipilimumab, which targets CTLA-4. The effectiveness of these immunotherapeutic agents in treating PCa has been demonstrated in several studies, but they do not benefit all PCa patients due to the highly heterogeneous nature of PCa [47, 48]. To improve treatment effectiveness, it is crucial to determine the molecular subtypes that can help predict which patients are most likely to benefit from immunotherapy.

In this study, we divided PCa into high-expression and low-expression clusters (cluster C1 and cluster C2) based on the expression levels of 65 marker genes highly correlated with macrophages. In subsequent studies, we found that cluster C2 has more active immune functions, higher infiltration abundance of all immune cells, higher expression of HLA and immune checkpoint genes, as well as higher stromal, immune, and estimate scores, demonstrating that C2 has more active TIME and is more likely to benefit from immune therapy compared to cluster C1. The more sensitive response of cluster C2 to PD-1 and CTLA-4 inhibitors in subsequent studies of this study proves the above point. Therefore, the TAMs-related molecular subtypes constructed in this study have the potential to identify populations that can benefit more from immunotherapy, so as to develop personalized treatment regimens and prolong the survival of PCa patients.

Furthermore, it has been found that TAMs may directly or indirectly participate in tumor proliferation and differentiation. Based on our analysis of intercellular communication, we found that TAMs are extensively involved in interactions with other cells through ligand-receptor pairs. Of note, TAMs interact with other cells in the TME of PCa primarily through MIF - (CD74+CXCR4) and MIF - (CD74+CD44) ligand-receptor pairs, providing new insights for the development of targeted therapies against TAMs.

The current risk grading system of PCa is mainly based on PSA, ISUP, and TNM staging to categorize patients into low-, intermediate-, and high-risk groups for different treatment choices [49, 50]. Traditional risk models, which primarily classify PCa patients based on their clinicopathologic features, have limited ability to assess the efficacy of emerging therapies such as immunotherapy and targeted therapy at the molecular level [51]. Therefore, in this study, we combined TAMs-related molecular subtypes with PCa prognosis information and constructed MRS consisting of 8 genes (ADAMTS14, LCN2, SCARA5, SYT4, NCF4, CHST13, FEV, PAX1). Among them, the high-risk group had worse PFS (p<0.001), with AUCs of 0.771, 0.772, and 0.741 for 1-year, 3-year, and 5-year, respectively, which had better predictive efficacy than traditional predictors, including PSA (AUC=0.509), ISUP staging (AUC=0.729), T staging (AUC=0.649), and N staging (AUC= 0.556).

Immunotherapy fights against tumors by utilizing the body’s immune system, and different tumors respond differently to immunotherapy due to differences in their TIME [52]. PCa is usually considered an immune ‘cold tumor’ with low tumor burden (TMB) and complex TME, which limits the benefits of immunotherapy [53, 54]. However, recent studies have shown that the TME undergoes dynamic changes, and with the changes in the TME, ‘cold tumor’ can also transform into ‘hot tumors’ [43]. As an inert tumor, PCa allows sufficient time for anti-tumor immunity to develop, so PCa, which has long been considered a cold tumor, also has the potential to benefit from immunotherapy. A previous study based on TAMs in HCC showed that the high-risk group had a higher TMB and lower levels of immune infiltration, indicating that patients in the high-risk group may be generally immunosuppressed and more susceptible to immune escape, making it more difficult for them to benefit from immunotherapy [45]. However, in PCa, through the MRS prognostic model established in this study, we found that the high-risk group had higher immune cell infiltration, higher expression of immune-related molecules, and lower TIDE scores, indicating that the TIME of patients in the high-risk group exhibited a phenotype of hot tumors, which make them more likely to respond to immunotherapy.

In this study, we identified NCF4 as the core gene in MRS. NCF4 is a type of NADPH oxidase complex that is involved in the production of extracellular reactive oxygen species (ROS). ROS induces the transformation of macrophages into TAMs in the TIME, thereby promoting tumor proliferation [55, 56]. Ryan et al. found that the downregulation and functional attenuation of NCF4 is associated with an increased risk of colorectal cancer progression, and colorectal cancer patients with high expression of NCF4 have a better prognosis [57]. Lee et al. also showed that NCF4 is associated with the risk of breast cancer incidence [58]. Chen et al. also demonstrated that high expression of NCF4 is related to poor prognosis in clear cell renal cell carcinoma patients, and knocking down NCF4 can inhibit the proliferation and migration of renal cancer cells [59]. In this study, we found that NCF4 is primarily expressed in macrophages within the PCa tissue, and the degree of macrophage infiltration in the TME is significantly positively correlated with NCF4 expression. Additionally, we also found that PCa patients with high expression of NCF4 have a worse prognosis. Currently, there is insufficient research on the association between NCF4 and PCa, which requires further exploration.

The study objectives were to identify macrophage-related molecular subtypes in PCa patients, establish an MRS model, and characterize the immune profiles between different molecular subtypes and between different risk groups. We have conducted multidimensional and multi-database validations, and the MRS model presents promising prospects for predicting the prognosis of PCa patients. There are, however, some limitations to our study. Firstly, this study is retrospective, and the data and corresponding clinical information are acquired from publicly accessible databases. The sample size is limited, and the analysis of clinical and pathological parameters is not comprehensive, which may lead to potential biases. Secondly, the role of NCF4 in PCa is unclear. In future research, the key gene NCF4 will be further explored through phenotypic and molecular biology experiments to determine its functional role in PCa.

In conclusion, this study identified TAMs-related genes in PCa patients, established and validated an MRS model to predict the prognosis of PCa patients, demonstrating good predictive ability, and evaluated the differences in TIME and immune therapy response among MRS risk groups. These results may help us further understand the characteristic changes of macrophage infiltration in PCa and provide new strategies for personalized treatment.

## Supplementary Material

Supplementary Figures

Supplementary Table 1

Supplementary Table 2

Supplementary Table 3

Supplementary Table 4

Supplementary Table 5

Supplementary Table 6

Supplementary Table 7

Supplementary Table 8

Supplementary Table 9
