# Supplementary material for: Comprehensive analysis of macrophage-related genes in prostate cancer by integrated analysis of single-cell and bulk RNA sequencing
Source: Aging (Albany NY). 2024 Apr 24;16(8):6809–38. doi: 10.18632/aging.205727 (PMC11087116; doi:10.18632/aging.205727)
Supplement: Supplementary Figures [file aging-16-205727-s001.pdf]

SUPPLEMENTARY FIGURES

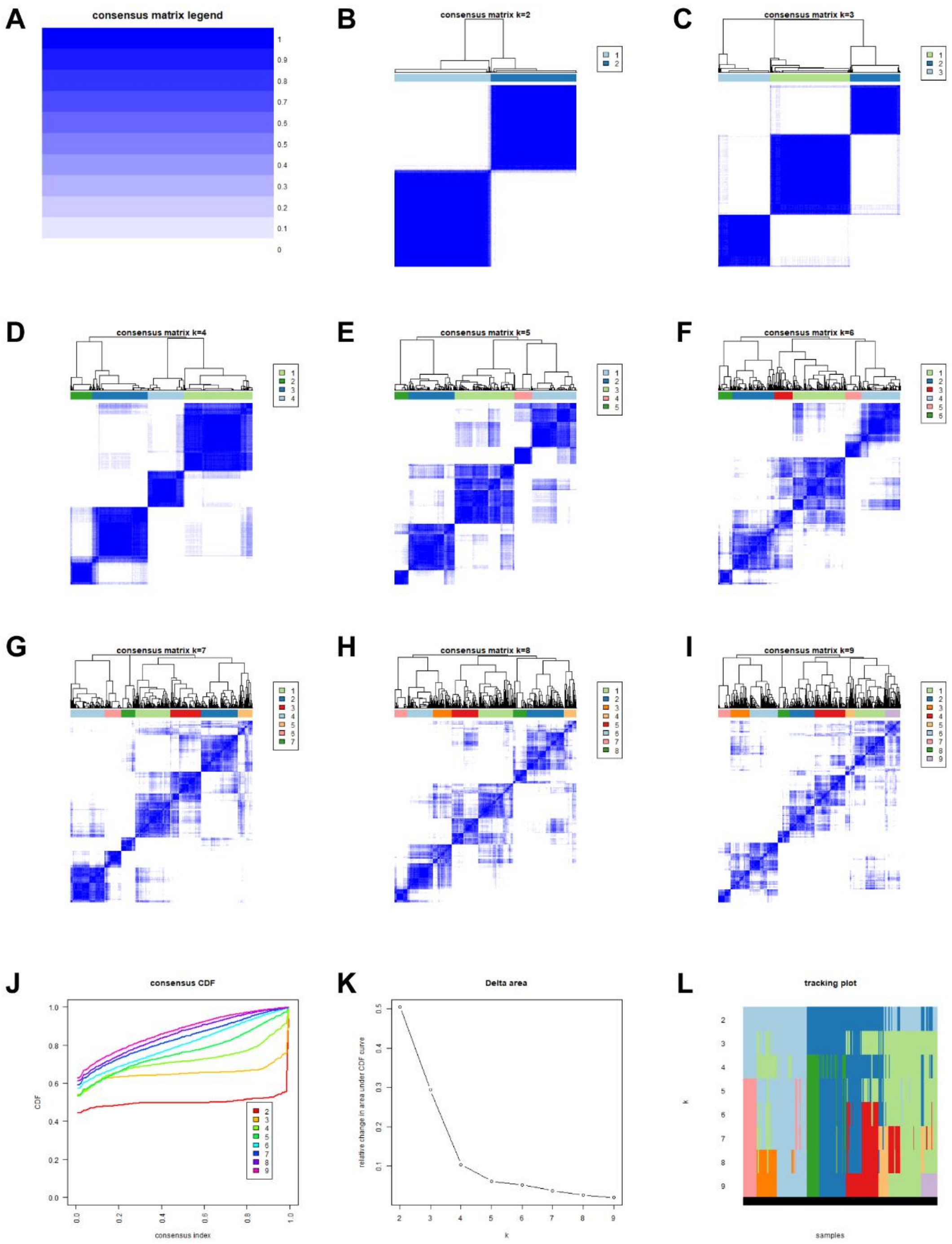

Supplementary Figure 1. (**A**) The legend of the consensus matrix. (**B**–**I**) Consensus clustering matrix when k = 2-9. (**J**) Consensus clustering CDF with k valued 2 to 9. (**K**) Relative change in area under CDF curve for k = 2 to 9. (**L**) Item tracking plot.

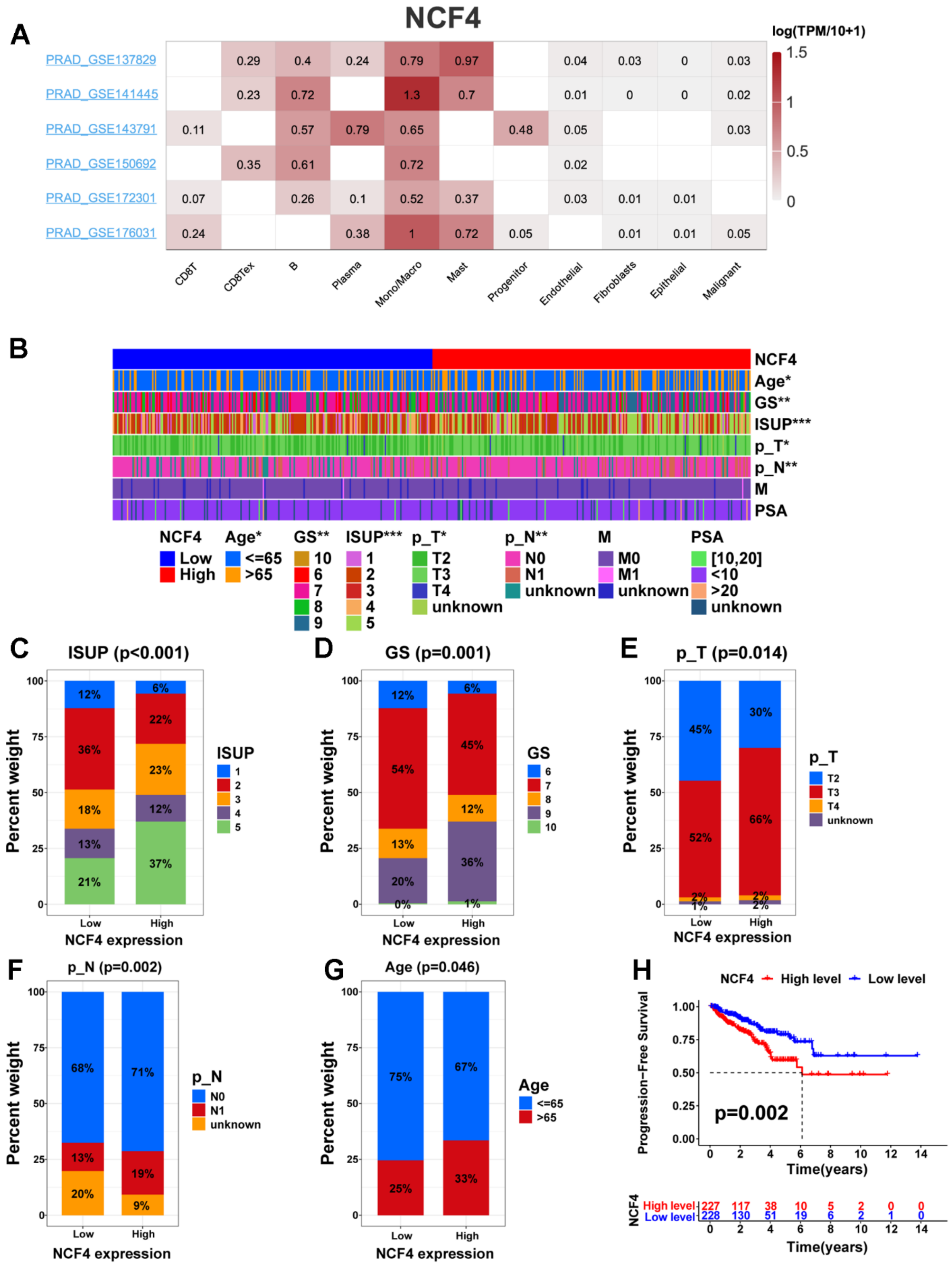

Supplementary Figure 2. (**A**) Utilizing the TISCH database to identify the expression patterns of NCF4 in immune and nonimmune cells across all six PRAD single-cell datasets. (**B**) Heatmap of NCF4 expression and clinicopathologic characteristics. (**C**–**G**) Relationship between ISUP, GS, p_T stage, p_N stage, Age, and the NCF4 expression. (**H**) Prognostic comparison between NCF4 high- and low-expression groups.
