# Supplementary material for: Comprehensive analysis of macrophage-related genes in prostate cancer by integrated analysis of single-cell and bulk RNA sequencing
Source: Aging (Albany NY). 2024 Apr 24;16(8):6809–38. doi: 10.18632/aging.205727 (PMC11087116; doi:10.18632/aging.205727)
Supplement: Supplementary Table 1 [file aging-16-205727-s002.pdf]

## SUPPLEMENTARY TABLE

**Supplementary Table 1. Detailed information on the PCa cohort used in this study.**

| Datasets | Platform | Number of input samples(tumor/normal) | Application |
|---|---|---|---|
| GSE141445 | GPL24676 Illumina NovaSeq 6000 (Homo sapiens) | 12/- | Identification of Macrophage-Cell Marker Genes |
| TCGA-PRAD | Illumina HumanHT-12 V4.0 expression beadchip | 501/52 | WGCNA, Molecular subtypes, Construction of the prognostic signature,… |
| CPGEA | Illumina HiSeq X TEN | 136/136 | WGCNA, External validation of the signature |
| DKFZ-PRAD | Illumina HumanHT-12 V3.0 expression beadchip | 81/- | External validation of the signature |
| MSKCC-PRAD | Affymetrix Human Exon 1.0 ST Array | 140/- | External validation of the signature |
| GSE116918 | GPL25318 [ADXPCv1a520642] Almac Diagnostics Prostate Disease Specific Array (DSA) | 248/- | External validation of the signature |
| GSE70768 | GPL10558 Illumina HumanHT-12 V4.0 expression beadchip | 126/- | WGCNA, External validation of the signature |
| GSE70769 | GPL10558 Illumina HumanHT-12 V4.0 expression beadchip | 92/- | External validation of the signature |
| GSE46602 | GPL570 [HG-U133_Plus_2] Affymetrix Human Genome U133 Plus 2.0 Array | 36/- | External validation of the signature |
| GSE70770 | GPL10558 Illumina HumanHT-12 V4.0 expression beadchip | 203/- | External validation of the signature |
