# Supplementary material for: Comprehensive analysis of macrophage-related genes in prostate cancer by integrated analysis of single-cell and bulk RNA sequencing
Source: Aging (Albany NY). 2024 Apr 24;16(8):6809–38. doi: 10.18632/aging.205727 (PMC11087116; doi:10.18632/aging.205727)
Supplement: Supplementary Table 7 [file aging-16-205727-s008.pdf]

Supplementary Table 7. The 65 intersection genes.

GMFG
HLA-DPA1
CTSZ
APOE
APOC1
C3AR1
EVI2B
STX11
HLA-DRA
LGALS9
LY96
FCER1G
TMEM176A
FCGR2A
HCLS1
ARHGDIB
C1QC
C1QA
C1orf162
CD53
TNFSF13B
TYROBP
LST1
ARPC1B
HLA-B
FXYD5
HLA-DPB1
VSIG4
HCST
AIF1
CD4
IFI30
CD74
GPNMB
CD48
SPI1
CTSS
TMEM176B
PLAU
PLEK
CD163
CSF1R
ADAP2
SLCO2B1
CD37
CPVL
ITGB2
MS4A6A

RNASE6
IGSF6
HLA-DQB1
HLA-DQA2
CYBB
HLA-DQA1
TYMP
UCP2
CYBA
TMSB4X
LAPTM5
CD14
C1QB
ALOX5AP
HLA-DMA
FCGR2B
LY86
