# Supplementary material for: Comprehensive analysis of macrophage-related genes in prostate cancer by integrated analysis of single-cell and bulk RNA sequencing
Source: Aging (Albany NY). 2024 Apr 24;16(8):6809–38. doi: 10.18632/aging.205727 (PMC11087116; doi:10.18632/aging.205727)
Supplement: Supplementary Table 9 [file aging-16-205727-s010.pdf]

Supplementary Table 9. The result of multivariate Cox regression analysis.

Model genes	Coefficient
ADAMTS14	0.515638204
LCN2	-0.154605129
SCARA5	-0.434633801
SYT4	0.135870367
NCF4	0.42349482
CHST13	0.664288472
FEV	-0.134764099
PAX1	0.204530723
